# Myalgic Encephalomyelitis/Chronic Fatigue Syndrome: A Comprehensive Review

**DOI:** 10.3390/diagnostics9030091

**Published:** 2019-08-07

**Authors:** Mateo Cortes Rivera, Claudio Mastronardi, Claudia T. Silva-Aldana, Mauricio Arcos-Burgos, Brett A. Lidbury

**Affiliations:** 1 Facultad de Medicina, Grupo de Investigación Neuros, Universidad del Rosario, Bogotá 110211, Colombia; 2 INPAC Research Group, Fundación Universitaria Sanitas, Bogotá 110211, Colombia; 3 Center for Research in Genetics and Genomics-CIGGUR, GENIUROS Research Group, School of Medicine and Health Sciences, Universidad del Rosario, Bogotá 110211, Colombia; 4 Group de Investigación en Psiquiatría (GIPSI), Instituto de Investigaciones Médicas, Facultad de Medicina, Universidad de Antioquia, Medellín 050002, Colombia; 5 The National Centre for Epidemiology and Population Health, RSPH, College of Health and Medicine, The Australian National University, Canberra ACT 2601, Australia

**Keywords:** immunological, chronic fatigue syndrome, myalgic encephalomyelitis, biomarker, neuroimmune, Epstein Barr virus, hypothalamic–pituitary–adrenal axis

## Abstract

Myalgic encephalomyelitis/chronic fatigue syndrome (ME/CFS) is a debilitating chronic disease of unknown aetiology that is recognized by the World Health Organization (WHO) and the United States Center for Disease Control and Prevention (US CDC) as a disorder of the brain. The disease predominantly affects adults, with a peak age of onset of between 20 and 45 years with a female to male ratio of 3:1. Although the clinical features of the disease have been well established within diagnostic criteria, the diagnosis of ME/CFS is still of exclusion, meaning that other medical conditions must be ruled out. The pathophysiological mechanisms are unclear but the neuro-immuno-endocrinological pattern of CFS patients gleaned from various studies indicates that these three pillars may be the key point to understand the complexity of the disease. At the moment, there are no specific pharmacological therapies to treat the disease, but several studies’ aims and therapeutic approaches have been described in order to benefit patients’ prognosis, symptomatology relief, and the recovery of pre-existing function. This review presents a pathophysiological approach to understanding the essential concepts of ME/CFS, with an emphasis on the population, clinical, and genetic concepts associated with ME/CFS.

## 1. Introduction

Myalgic encephalomyelitis/chronic fatigue syndrome (ME/CFS) is a disabling clinical condition characterized by unexplained and persistent post exertional fatigue accompanied by a variety of symptoms related to cognitive, immunological, endocrinological, and autonomous dysfunction [1,2]. The estimated prevalence is estimated at 0.1–0.5% [3,4]. As a result of this debilitating condition, the burden for patients and caregivers is tremendous. In a recent review of the ME/CFS literature reported by The Institute of Medicine (IOM) of the United States (US), it was estimated that between 836,000 and 2.5 million Americans suffer from ME/CFS, causing an annual financial cost that ranges between 17–24 billion (USD) dollars per year [5]. The individual income losses are approximately $20,000 annually per household, and the unemployment rates among those who suffer this pathological condition are between 35–69% [5]. ME/CFS constitutes a particular enigmatic, debilitating and costly significant public health problem [6]. It is characterized by a substantial reduction in previous levels of occupational, educational, social and personal activities in a patient’s lifetime. The disease affects all ages, races and socioeconomic groups and some studies showed that approximately three to four times as many women as men present the symptoms [3,7,8]. Table 1 shows the role of the main tissues involved in the pathogenesis of the disease following the hypothesis of the 3 pillars, explained later in the text.

## 2. History

In the World Health Organization (WHO) International Classification of Diseases version 2016, both ME and CFS were coded identically and classified as other disorders of the nervous system; nevertheless, “fatigue syndrome”, which non-expert clinicians may view as synonymous with CFS, is classified under mental and behavioral disorders [15]. This leads to confusion in the classification of the aetiology of the disease, mainly for primary care physicians.

Of the two current definitions, myalgic encephalomyelitis (ME) was the first to be defined. In 1934, multiple cases of an unknown illness were recorded around the world. The cases were confused with poliomyelitis or other medullary diseases, but it was eventually differentiated and first known as “neuromyasthenia” [5], with symptom attribution to psychological causes. The details changed from each patient, but in general, patients experienced a variety of symptoms including malaise, tender lymph nodes, sore throat, pain, and signs of encephalomyelitis [16]. As it occurs presently, the aetiology could not be clearly determined, and it was highly suspected to be infectious because of the flu-like prodrome in most of the affected patients. In 1959, the term “benign myalgic encephalomyelitis” eventually was chosen to reflect an inflammatory disease characterized by severe muscular pains and the evidence of parenchymal damage to the nervous system in the absence of mortality [5].

The landmark case from this era occurred in 1955, and became known as “Royal Free disease” through its association with an English hospital of the same name. Fifty-five nurses, doctors, assistants, and other health personnel were hospitalized on presenting a series of symptoms, which was unusual for the time. Interestingly, most of this hospitalized group contracted upper airway infection prior to the onset of the disease, as well as gastrointestinal alterations, acute vertigo and sore throat, which were followed thereafter by severe headache accentuated by movement and change of position, nuchal pain, pain in the limbs, extreme lassitude, and paranesthesia. Some cases reported more critical symptoms, including muscular cramps and twitching, objective sensory impairments, muscle tenderness, cranial nerve palsies, and ocular movement disorders, suggesting “epidemic neuromyasthenia” [17]. From that point, the efforts to look for the aetiology and the treatment of “myasthenia” began to grow.

In the 1970s, the European psychiatric society proposed that myalgic encephalomyelitis (ME) was a psychosocial phenomenon caused by either mass hysteria or the altered medical perception of the community, renaming the disease to “myalgia nervosa”. With this redefinition and no organic explanations of the disease, the medical community began to consider the psychiatric component to understand the condition. This perception among medical professionals vastly limited the research efforts to study ME in fields different from psychiatry and psychology [5,18]. Later on, some researchers demonstrated the severe long-term disability of the disease and abolished the term “benign.” [15].

In 1986, Ramsay [17] published the first diagnostic criteria for myalgic encephalomyelitis, which is a condition characterized by a unique and chronic form of muscle fatigability even after a minor degree of physical effort, spending three or more days to restore full muscle power. At the end of the 1980s, two cases of an illness resembling mononucleosis attracted the attention of some medical communities [19]. The disease was then defined as “chronic or recurrent debilitating fatigue and various combinations of other symptoms, including a sore throat, lymph node pain and tenderness, headache, myalgia, and arthralgia” [5]. Since this time, the illness was largely linked with Epstein-Barr virus (EBV) infection onset and was known as “chronic Epstein-Barr virus syndrome”.

In 1987, the US Center for Disease Control and Prevention (CDC) convened a working group to reach a consensus on the clinical criteria of the disease. After much debate about the disease nomenclature, the CDC reached the consensus of “chronic fatigue syndrome”, but noticed that the term “myalgic encephalomyelitis” was the name that was most accepted in other parts of the world. That was the origin of ME/CFS, the term for this condition, that is currently accepted worldwide [15,20]. In 1994 Fukuda proposed a clinical and investigative protocol in order to recreate a comprehensive and integrated approach to study ME/CFS.

The definition that was proposed by Fukuda considers chronic fatigue as “self-reported persistent or relapsing fatigue lasting six or more consecutive months” and requires a clinical evaluation to identify or rule out other medical or psychological conditions that may explain the symptomatology [21]. A diagnosis of ME/CFS involves the absence of other fatigue-associated conditions, unexplained fatigue for at least six months, and at least four of eight minor symptoms. Although this definition had been widely criticized for being overly inclusive, it is still used in the clinical evaluation and diagnosis of the disease. As well as the 1994 criteria, up to 20 other clinical criteria have emerged [1], with the other notable clinical consensus criteria being the 2003 Canadian Criteria, which was an update in 2011–12 to the International Consensus Criteria [2,22].

ME was recognized by the World Health Organization (WHO) in 1969 as a defined organic neurological disorder. However, in the daily medical practice globally, the concept of ME was not well recognized. The disease formerly described as ‘epidemic neuromyasthenia’ in the US is now more likely to be diagnosed as Chronic Fatigue Syndrome (CFS). Unfortunately, it was not until the ICD-10 that CFS was included in the medical classification of diseases in the US, with inclusions such as benign myalgic encephalomyelitis and post-viral fatigue syndrome [23]. Also, as a prolonged atypical illness associated with serological evidence of a persistent Epstein-Barr infection, it was associated with infectious mononucleosis syndrome [24]. However, six years later, Holmes described the first combination of nonspecific symptoms of the syndrome, introducing major and minor criteria for the ME/CFS in the clinical practice [25].

Since the definition of this syndrome, the main issue to diagnose the condition has been the absence of objective parameters to facilitate an accurate clinical assessment of the patient. Patients with ME/CFS were frequently maligned and told they did not have a real physical illness, but rather a psychological condition [26]. Through years of molecular research and clinical investigation, several clinical definitions have been established in the literature. However, the most widely used in clinical trials have been the Fukuda criteria and the international criteria, both with an inability to separate the ME from the CFS [26,27]. Therefore, a case of ME/CFS is defined by the presence of an unexplained, persistent, and relapsing chronic fatigue of new onset that is not alleviated by rest, which results in a significant reduction in the quality of life, and a concurrent occurrence of four or more of the following eight symptoms that must have persisted during six or more consecutive months:

1. “Brain fog” described as impairment in short-term memory or concentration severe enough to cause a reduction in previous levels of personal activities; 2. A sore throat; 3. Tender cervical or axillary nodes; 4. Muscle pain; 5. Multipoint pain without joint swelling; 6. Headaches; 7. Unrefreshing sleep; 8. Post-exertional malaise lasting more than 24 h [21].

Even though over the last few decades clinicians have reached a consensus to diagnose ME/CFS, the underlying aetiology is not well understood [1]. It is noteworthy that prior to a ME/CFS diagnosis, patients are mostly healthy, fully functional and have a good quality of life. Approximately 50–80% of patients with ME/CFS start suddenly with a flu-like illness, from which patients do not recover. ME/CFS is commonly found after infection by a virus, bacteria, or parasite, suggesting an immunological dysfunction as the possible beginning of the multi-systemic impairment, which is accompanied by a strong genetic predisposition, as shown in a twin analysis [26,28].

There has been an increasing effort to investigate the aetiology and maintenance of symptoms, including for patients with no infectious prodrome. Life stressors are shown to cause a negative impact on the neuroendocrine circuits of stress, leading to other complications besides immunological impairment [26]. Stress can be triggered by inflammatory components. Interestingly, one of the hypotheses that attempts to explain the aetiological component of the condition suggests the involvement of inflammatory cascades impairing either the functionality of the blood–brain barrier or the gut barrier [6] with other studies indicating that infections and immunological dysfunction contribute to the development and maintenance of symptoms, probably interacting with genetic and psychosocial factors [1].

Viral involvement is a well-supported pathophysiological theory due to the high index of an infectious onset in at least half of the patients, and confirmed findings of biochemical dysregulation of the 2-5A synthetase/ribonuclease L antiviral defense pathway in monocytes [2]. The alteration of this pathway and the reduced cytotoxic activity of the NK cells in ME/CFS patients are recognized as the main findings of the immunological impairment on ME/CFS patients [29]. In addition to immune and viral aetiology, the attenuation of the hypothalamus–pituitary–adrenal (HPA) axis is documented in adult and adolescent ME/CFS patients, with marked evidence of systemic hypocortisolism, which is an aspect that can influence the immunological and neuronal homeostasis of the individual [30]. Also, previous studies have reported enhanced sympathetic nervous activity, as well as increased levels of catecholamines in ME/CFS, evidencing a neuroactive pattern in the pathophysiology of the syndrome [31]. The alterations of these three biological systems will be further discussed.

The purpose of this review is to propose a coherent connection between the three pillars of the pathophysiology of the ME/CFS described in the literature: the immunological system, the neuroendocrine system, and the central nervous system, leading to a clear explanation of the symptomatology of the disease in the absence of a reference standard.

## 3. Epidemiology

The prevalence of ME/CFS varies among different studies depending on how this pathological condition is defined, the population surveyed, and the employed methodology. Terminological variations and inconsistencies in how definition and labels are used in different studies of ME/CFS pose a challenge to compare different global cohorts [32]. Therefore, there is a need to unify the diagnostic terminology, and this constitutes a goal in the investigation process. 

Fatigue is commonly experienced by patients presenting different pathological conditions. Thus, it is necessary that any clinical measurement of fatigue differentiate between ordinary fatigue and a symptom of a pathological disease [33]. It is essential to be aware that the boundaries between normal and abnormal fatigue are arbitrary. For instance, some authors argued that fatigue should be considered unusual when the fatigued person views himdelf or herself to be ill [34,35]. A number of surveys conducted around the world proposed that the prevalence of fatigue among the adult population is considerable. The Office of the Privacy Commissioner of Canada (OPC) survey of psychiatric morbidity in the United Kingdom (UK) found that 27% of all adults reported significant fatigue in an ordinary week, and pointed out that the prevalence of chronic fatigue was 13.4% in that population [36]. A comparable study from the United States reported a prevalence of 6% of unexplained fatigue lasting more than two weeks [37,38]. One study involving the population of 143,000 people between 18–64 years of age from England reported an incidence of 4.7% of the included cases in the population and an overall prevalence rate of 0.20% [33]. Lawrie et al. estimated the annual incidence of CFS as 370 per 100,000 and the prevalence as 740 per 100,000 individuals in a study executed in Edinburgh, Scotland [39]. 

ME/CFS is an endemic disorder that affects all racial/ethnic groups and is seen across all socioeconomic strata. Most studies of gender differences report higher rates in women [8]. However, it has been suggested that gender differences may occur as an artefact of recruiting samples in a gender-biased manner from different specialist centers. For example, in a prospective cross-sectional cohort study performed in a rheumatology center in Spain, statistical tests were omitted to determine the significance of the gender, suggesting a possible bias [40]. Nonetheless, many community-based studies indicate that there may be a real gender difference [41,42].

The median duration of the illness is approximately seven years, a quarter of those with the disease are unemployed or disabled, and the average affected family forgoes approximately $20,000 in annual earnings and wages [43]. Kroenke et al. found a comparison with the disability experience in some medical conditions such as untreated hyperthyroidism and myocardial infarction [44].

The prognosis of patients with ME/CFS is variable. For some patients, an improvement in symptoms is a more commonly reported outcome than full recovery, and the prognosis in this case is less disappointing, especially among patients in primary care [45]. Some of the most prominent risk factors that predict progression are the severity of the symptoms at the time of onset, the standard of early management of the disease, having a mother with the syndrome, and comorbid diagnosis of fibromyalgia. The attribution of CFS to a physical cause and poor control over the symptoms have been associated with worse outcomes in those patients [46]. Death in patients with ME/CFS is usually caused by another co-existing illness, in which cancer and cardiovascular abnormalities are the most common causes of death among this population [47,48].

## 4. Clinical Manifestations

Myalgic encephalomyelitis/chronic fatigue syndrome (ME/CFS) is a complex condition with multiple systemic dysfunction. The primary symptom is the post-exertional fatigue accompanied by various neurological, cardiovascular, respiratory, and gastrointestinal manifestations [49]. Although an attempt was made to systematize the clinical signs of the disease through some major and minor criteria, clinical heterogeneity is far from being covered. However, most of the clinical trials accept and use the Fukuda and/or the CDC criteria for patient selection. Research efforts continue seeking biomarkers to aid aetiological understanding, clinical selection, and treatment options for this debilitating condition [26]. 

In order to make the diagnosis of ME/CFS, the patients must have the following criteria shown in Table 2 for at least six months in adults, and three months in children.

Other unspecific symptoms also reported by patients include dizziness, nausea, anorexia, headaches, and night sweats [49]. The onset of the disease may vary depending on demographic factors. For instance, most patients that attend tertiary care health medical doctors frequently report an acute onset of symptoms after an infectious illness; nevertheless, in the general population attending primary care, the onset of fatigue is gradual [53,54].

The fatigue in patients suffering ME/CFS is more intense and different from usual tiredness related to physical exercise. It may combine cognitive and physical exhaustion, weakness, heaviness, general malaise, light headedness, and sleepiness. These characteristics are the main tool that the primary care physician has to be able to differentiate ME/CFS from other common causes of fatigue, including fatigue associated with cancer.

Post-exertional malaise (PEM) is considered one of the distinguishing symptoms of ME/CFS. It can be used by the clinicians to differentiate it from other conditions with similar symptomatology such as depression. PEM refers to severe fatigue after minimal physical or mental/cognitive exertion. The mental fatigue is described by the patients as “brain fog” and includes poor concentration, forgetting words in speech and poor short-term memory [54]. 

The autonomic dysfunction may elicit a central alteration of the autonomic nervous system, which provides unconscious control of basic systemic functions [49]. The symptoms of the autonomic dysfunction include dizziness and fainting upon standing up, inability to alter heart rate with exercise, sweating abnormalities, digestion difficulties due to slow digestion, and urinary, sexual and visual problems [54]. This autonomic dysfunction is related to one of the most common comorbidities found in the syndrome, the Postural Orthostatic Tachycardia Syndrome (POTS), which is defined by Reynolds et al. as the presence of orthostatic intolerance and an increased heart rate of more than 30 beats per minute from baseline [53].

All of these signs are suggestive of ME/CFS, but are not specific. Patients feel tired; they may have drooped and crooked postures with rounded shoulders when sitting in a chair. Some patients need to lie down on the examination table while they wait to be seen by the physician; others go to the hospital in a wheelchair [26]. Some patients may present periorbital hyperpigmentation indicating allergies, and puffy eyes, meaning fatigue or oedema. Patients with a sore throat are more likely to report a viral reactivation accompanied by tender lymphadenopathy in the cervical, axillary and inguinal areas [54]. Examination of the pupils may show oscillation of the pupils or diminished pupillary accommodation due to the imbalance of the central sympathetic and parasympathetic nervous system [55].

## 5. Pathophysiology

### 5.1. Immune System

The pathophysiological mechanism of ME/CFS is unclear [56]. In the context of the immune system, hypotheses include altered central nervous system functioning resulting from abnormal responses to common antigens; the activation of inflammatory, cell-mediated immune (CMI) response, and enhancement of oxidative and nitrosative pathways; a neuroendocrine disturbance, and autoimmune responses against neuronal and other cells and proteins [29].

Numerous studies have sought evidence for a disturbance in the immunity system. A decreased function of natural killer (NK) cells, an alteration in cytokine profile, and the reduced responses of T cell to mitogens, and other specific antigens, have been reported in several studies [56]. The immunological findings encompass a low-grade inflammation, as indicated by an increased production of nuclear factor kB (NF-κB), cyclooxygenase-2 (COX-2), and inducible nitric oxide synthase (iNOS); immune activation, with increased expression of activation markers; increased levels of proinflammatory cytokines, including IL-1, IL-4, IL-5, IL-6, and IL-12 and lowered levels of IL-8, IL-13, and IL-15; immunosuppression, as indicated in decreased NK cell cytotoxicity; autoimmune reactions; oxidative and nitrosative stress (O&NS) damage to membrane fatty acids, proteins and DNA; lowered antioxidant levels; mitochondrial dysfunction; bacterial translocation, and the alteration of antiviral response elements, such as the 2-5 oligoadenylate synthetase/RNase L pathway [29,56,57].

### 5.2. Inflammation and Oxidative Stress

Studies have reported that ME/CFS is accompanied by systemic inflammation, while others define ME/CFS as a low-grade inflammatory disease characterized by: (a) increased levels of pro-inflammatory cytokines and high concentrations of acute phase reactants; (b) diminished levels of antioxidants, including zinc and coenzyme Q10 and antioxidant enzymes; (c) O&NS damage to fatty acids, proteins and DNA; (d) dysfunctional mitochondria; (e) a lowered w3/w9 polyunsaturated fatty acid ratio; (f) increased translocation of Gram-negative bacteria; and (g) aberrations in intracellular signal transduction and apoptosis pathways [58].

The high level of pro-inflammatory cytokines is an important feature of the pathophysiology and may explain some of the clinical manifestations of the disease, such as chronic fatigue and flu-like symptoms [56]. The most supported origin of this inflammatory state is the association with an infectious pathogen, especially a viral infection. A viral infection could rapidly activate antiviral pathways, e.g., IFN-γ-indicated pathways, which would quickly allow the establishment of a systemic inflammatory state [59]. 

The evidence that immunoinflammatory pathways are activated are:

(a) chronic low-grade inflammation, as indicated by elevated production or levels of tumor necrosis factor-alpha (TNF-α) and other pro-inflammatory cytokines such as IL-1β and IL-6;
(b) Cellular-mediated immune (CMI) response activation, as indicated by increased neopterin levels, a well-known biomarker of the immunological stimulation;
(c) immunological phenotypic change including Th1 to Th2 shift, increased CD26 expression on T cells, defective T regulatory cell functions, and T cell exhaustion [11,60,61]; and
(d) increased bacterial translocation sustained by the leaky gut theory of increased IgA levels against lipopolysaccharides (LPS) of Gram-negative bacteria [29,58].

Oxidative stress is defined as a disturbance to the equilibrium status of pro-oxidant and antioxidant systems in favor of pro-oxidation. The term is used to describe chemical reactions involved in the production of free radicals and other reactive molecules that potentially induce cellular injury [62]. 

ME/CFS patients have a significant activation of the oxidative and nitrosative stress, which appears to be a critical feature in the pathophysiology of the disease; for example, studies have reported the elevation of oxidative stress biomarkers in blood (notably isoprostane, oxidized LDL, and iso-prostaglandin F2), and the reduced antioxidant capacity as represented by glutathione levels [62,63]. These findings suggest that oxidative stress could be implicated in the pathophysiology of ME/CFS by an excessive free radical formation, but not by the depletion of antioxidant reserves [58,62]. The damaging effects of the O&NS to fatty acids or proteins transform them into immunogenic targets for the immune system as it loses immunogenic tolerance [59,64]. This mechanism might explain the high incidence of IgM-mediated autoimmune responses directed against O&NS-modified epitopes in ME/CFS.

An important aspect that allows the integration of the immunological phenomena is the relationship between mitochondrial damage and the increase of oxidative stress [64]. Increased ROS leads to damage of the electron transport chain leading to depleted adenosine-5′-triphosphate (ATP) production which in turn causes a deficiency in oxidative phosphorylation and impaired mitochondrial function [62,65]. Mitochondrial aetiology for ME/CFS is a prominent feature of current thinking, and whether immune dysregulation explains all aspects is yet to be determined [66,67,68].

Viral infections, including ROS/RNS-induced damage and inflammatory cytokines can activate a key transcription factor NF-κB, which could play a triggering role and propel an inflammatory cascade in patients. Once activated, NF-κB is translocated from the cytoplasm to the nucleus to bind the DNA promoter sequences of several inflammatory mediators such as IL-1β, IL-6, TNFα; and O&NS mediators, such as cyclooxygenase-2 (COX-2) and inducible NO synthase (iNOS) [64,69]. This upregulation could link the interaction between the increase in oxidative stress and the immunological overreaction in these patients. However, the directionality of this interaction is not clear yet. It is believed that the immune response to an external pathogen could be the cause of the activation of unbridled oxidative stress, which in turn perpetuates a steady inflammatory cascade [59,70].

### 5.3. NK Alteration

The NK cells are granular lymphocytes that play an important role at the interface between innate and adaptive immunity [29,71]. NK cells are important effectors of the innate immune system, as they responsible for the lysis of tumor and virally infected cells without affecting healthy host cells. [72]. The surface markers in NK cells indicate different subtypes with different functions. For example, two subsets of NK population known as CD56^bright^ CD16^dim/^^−^ and CD56^dim^ CD16+ have different functions [73]. The former subset plays a predominately immunosurveillance role with potent cytokine production, whereas the latter subset is primarily cytotoxic and can kill infected and tumor cells [29,74]. CD16, or FcyRIIIA, is a low-affinity receptor for the Fc portion of immunoglobulin G, and CD56 is an adhesion molecule that mediates the homotypic adhesion in immunological cells [75]. For the proper interaction with the cells, NK cells are recruited mainly by interferon and chemoattractive chemokines, including CCL22, CX3CL1, and CXCL8, with an alteration of intracellular Ca^+2^ concentration impacting lytic potential [76]. Decreased NK cell function and associations of NK impairment with viral infection/reactivation have been observed in ME/CFS patients.

A study examining 41 ME/CFS patients reported a decreased frequency of peripheral blood CD3-CD57+ lymphocytes; primarily representing NK cells, whereas frequencies of CD3+CD57+ cells, representing cytotoxic T lymphocytes, were unperturbed, suggesting an immunological selectivity in the disease [74,77,78]. However, another study found a significantly lower frequency of CD3+ lymphocytes and a higher CD4 T-cell representation in ME/CFS patients [79], whereas several studies found no significant changes in the immunological profile between ME/CFS patients and controls [80].

Transient receptor potential (TRP) ion channels are involved in diverse physiological processes, such as the sensation of a wide range of stimuli and modulation of ion entry to the cytoplasm [81]. Recently, the TRPM3 ion channel was shown to display a significantly lower expression on the cell surface of NK, and B cells form ME/CFS patients when compared with healthy controls. This finding suggests an impaired Ca^+2^ mobilization in the NK cells of ME/CFS patients, which prevents the mobilization of secretory vesicles leading to a reduction in NK cell cytotoxic activity [56,66]. Interestingly, one study recently demonstrated an alteration in the signaling of the MAPK pathway that was associated with a decrease of the intracellular concentrations of Ca^+2^ in NK isolated cells of patients with ME/CFS, suggesting a fundamental alteration in the lytic function of these immune cells [29]. Additional studies reported findings for a number of SNPs in genes for acetylcholine receptors (AChR) and TRP ion channels from the isolated NK cells from ME/CFS patients. These investigations reported a significant reduction in NK lysis in these patients compared with unfatigued controls [82].

Interestingly, other studies established that mitochondria play a key role in the function of innate immunity cells, including NK cells. A recent discovery that mitochondria express a range of AChR subtypes, including nicotinic α3 subunit receptor, suggests that nAChR may impact the mitochondrial function directly to regulate oxidative stress [29,75]. In the face of dysregulation in several neurotransmitters, including acetylcholine, the mitochondrial stress associated with the activation of these nicotinic receptors would lead to an alteration in intracellular dynamics in several immune cells, including NK cells.

### 5.4. Immunoglobulins

Levels of total serum immunoglobulin G (IgG) and the IgG subclass are reduced in CFS patients. Some studies report decreased IgG1 and IgG3 levels; others observed deficiencies in all IgG subclasses or only in the IgG3 levels in some patients. These deficiencies may be correlated with antiviral activity in these patients and contribute to the pathogenesis of the disease [83]. ME/CFS is accompanied by increased serum levels of IgA and, to a lesser extent, IgM against the LPS of commensal Gram-negative enterobacteria [84]. It is well known that persistent inflammation in the syndrome can cause the mucosal barrier to become more permeable, whereby wider spaces between the cells of the gut wall cause a loss of the protective barrier. This may induce increased bacterial translocation, and thus an increase of serum endotoxin concentrations, which might trigger an immune response [85]. The increased serum IgA and IgM levels against the LPS of the Gram-negative enterobacteria in ME/CFS indicate the presence of increased gut permeability and an immune response mounted against the LPS of the enterobacteria [83]. Although there are no exact figures to pathologize the levels of IgA in CFS, an elevation of this immunoglobulin can help the primary care physician establish the progress of systemic inflammation in the natural history of the disease.

### 5.5. Autoimmunity

Maes et al. reported in a study analyzing the IgM levels from the serum of ME/CFS patients, that the syndrome is characterized by an IgM-related immune response directed against disrupted lipid membrane components, by-products of lipid peroxidation, S-farnesyl-l-cysteine, and NO-modified amino acids, which are not usually detected by the immune system and, due to oxidative and nitrosative damage, have become immunogenic [58]. These findings suggest that an underlying infection may be present in these individuals, and that the immune system is chronically activated in response to a pathogen [56]. The onset of autoimmune responses in ME/CFS may be explained by different mechanisms, including the creation of new epitopes due to the effects of O&NS; inflammatory processes that produce a state of immunologic overreaction; and increased bacterial translocation, with new immunogenic molecules coming into contact with the immune system [86].

Recently, a subset of patients with ME/CFS was shown to display an array of autoantibodies directed against neurotransmitters and neuronal components including serotonin, anti-neural antibodies, gangliosides, and mu-opioid, dopamine D2, muscarinic, and 5-HT1A receptors [87]. Thus, the neurotransmitter alteration could explain many of the disease symptoms such as the neurocognitive dysfunction, sleep alterations, or even a central fatigue theory.

Anti-conjugated oleic, palmitic, and myristic acid, malondialdehyde, azelaic acid, and S-farnesyl-l-cysteine autoantibodies levels are significantly higher in ME/CFS patients than in normal control [58,59,88]. This increased autoantibody production presumably reflects cellular damage or breakdown, which represent increased autoantibody production to self-epitopes that became immunogenic due to oxidative stress [56]. ME/CFS is also accompanied by increased anti-conjugated NO^•^ adducts, e.g., NO-tyrosine, NO-phenylalanine, NO-arginine, NO-tryptophan [83]. The IgM responses to these conjugated NO-derivatives represent an autoimmune response to nitrosylated self-epitopes that became immunogenic due to nitrosative damage.

Several studies described autoantibodies in ME/CFS, mostly against nuclear and membrane structures and neurotransmitter receptors, as pointed out earlier. Antinuclear antibodies (ANA) were found between 13–68% of the ME/CFS patients, dsDNA antibodies were found in 12% of patients, further autoantibodies against endothelial and neuronal cells were described in 30% and 16% of patients, respectively [89].

Antibodies against the muscarinic M1 acetylcholine receptor (AChR) were reported in 15% of ME/CFS patients and were highly associated with muscle weakness and muscle pain [87,90]. Antibodies against B1 and B2 adrenergic receptors were found in 29% of patients with ME/CFS compared to healthy controls [91]. The association of B2 receptors autoantibodies with immune markers suggest an activation of B and T cells expressing B2 adrenoreceptors [92]. Disturbance of these receptors and M1 AChR function may explain the symptoms related to autonomic dysregulation, and some of the most common comorbidities of the syndrome, for example, postural orthostatic tachycardia syndrome (POTS) [53]. 

There is compelling evidence that all of the immunological alterations seen in the pathophysiology of ME/CFS are associated with autoimmunity, suggesting a possible hallmark for the understanding of the disease. Nowadays, the search for autoantibodies is of great importance allowing the development of potential biomarkers for the diagnosis of the disease and thus providing further advances for therapeutic interventions [93].

#### 5.5.1. B Cell Impairment

The profile of B cell subpopulations could be different in ME/CFS compared with controls. However, this variation is still not fully elucidated within the pathophysiology of the disease [94]. The constant interaction of B cells with an infectious pathogen leads to the dysfunction of the immunological tolerance, losing mechanisms that generally prevent the development of autoreactivity. New memory B cells with autoreactivity, which normally would be eliminated by the thymus, arise and persist in these individuals [13]. When the patient is exposed to a new infection, these B cells could produce antibodies that react both to microbe and autoantigens. This is the basis of the mimicry theory behind autoimmune disease [95]. Abundant studies have proved this theory with immunological biomarkers; ME/CFS is associated with a significant increase in the amount of B cell with a CD20^+^ CD5^+^ phenotype, which is correlated with autoantibody production and with overexpression of CD21 markers, which acts as a receptor for some viruses, including EBV [56,96].

#### 5.5.2. 5′-Oligoadenylate Synthetase/RNase L Pathway

The association between the onset of ME/CFS with a viral infection has always been a fundamental pillar in the understanding of the pathophysiology of the disease, as the viral exposition has been one of the most supported triggers of the disease. As a result, interest in the antiviral pathways in these individuals has increased widely, and its disturbance may explain the onset of the immunological dysfunction partially. One of the principal interferon-activated antiviral pathways involves the activation of the 2′-5′-oligoadenylate (2-5A) synthetase/RNase L system, which is an innate immunity pathway that responds to a pathogen-associated molecular pattern to induce the degradation of viral and cellular RNAs to block viral infections and propagation [97,98]. It is composed of three types of enzymatic activity: 2-5A synthetase, 2-5A degrading enzymes, and RNase L, which drive the antiviral and antiproliferative effects of type I interferons [98,99,100].

On a theoretical basis, some intracellular antigens are capable of deregulating the 2-5A synthetase/RNase L pathway in patients with ME/CFS [97]. Severe deregulation of the 2-5A synthetase/RNase L pathway is accompanied by the down-regulation of apoptotic activity in the peripheral blood mononuclear cells (PBMC) of patients due to the accumulation of proteolytic cleavage products. The initial up-regulation of apoptosis in these cells due to the alteration of the antiviral pathways is followed by a subsequent down-regulation [95]. Therefore, down-regulated apoptotic activity implicates a suppressed ability to eliminate intracellular antigens, similar to EBV or Mycoplasma spp. [99,101].

As well as triggering the 2-5A synthetase/RNase L activation, type I IFN induces the expression of protein kinase R (PKR) [97]. The activation of this enzyme, as typically seen during viral infection or cellular stress, results in a blockade of protein synthesis and consequent cell death through the activation of some transcription factors [102]. Recently, the role of PKR related to metabolism, inflammatory processes, cancer, and neurodegenerative diseases has gained interest because of its relevance; it does not act just as an antiviral agent, but also as a cell growth regulator [103]. However, there is conflicting data regarding the activity of PKR in ME/CFS patients, so the further investigation of PKR activity in CFS patients is warranted [101].

#### 5.5.3. Central Nervous System Alteration

##### Neuroinflammation

One of the reasons why ME/CFS is profoundly disabling is due to the neuropsychologic symptoms that patients experience, including cognitive impairment, decreased alertness, impaired memory and concentration, and depressive symptoms. In addition, they also experience widespread chronic pain, including headaches, muscle, and joint pain [104]. These findings suggest that the central nervous system (CNS) is deeply involved in the pathophysiology of the disease [105,106].

Previous studies with functional imaging showed hypoperfusion and a reduction of the biosynthesis of neurotransmitters such as glutamate, aspartate, and gamma aminobutyric acid (GABA) in the frontal, temporal, cingulate and occipital cortices and basal ganglia. Using single photon emission computed tomography (SPECT), some studies found ME/CFS-related perfusion defects in the frontal and temporal lobes and impaired cerebral blood flow [107,108]. A voxel-based morphometry study demonstrated volume reduction of the bilateral prefrontal cortices in ME/CFS patients, and the volume reduction level in the right prefrontal cortex was associated with the severity of fatigue and the reduced functional status [109]. The reaction time was slower and the amplitude of electroencephalography-derived premovement-related cortical potential was also reduced in CFS in comparison with healthy individuals [108]. In this section, we will explain the neurological dysfunction and the possible interactions with other systems in ME/CFS patients.

With pro-inflammatory activity recognized for ME/CFS, it has been suggested that neuroinflammation is involved in its pathogenesis and progression [104]. Neuroinflammation is evidenced in ME/CFS patients by the activation of glial cells, specifically microglia and astrocytes. The activated glia exhibits an increase in the expression of the 18-kDa translator protein (TSPO). This protein can be assessed by PET to ascertain the inflammatory activation within the CNS [105]. Conversely, this constant interaction between the CNS and the proinflammatory cytokines results in “sickness behavior”, which is a state that is characterized by malaise, lassitude, fatigue, numbness, reduced appetite, reduced social interactions, fatigue, and weight loss, which is similar in many aspects to major depression [110,111]. Additionally, Hornig et al. demonstrated a significant increase in the levels of proinflammatory cytokines in the cerebrospinal fluid of patients with CFS/ME. They made a network analysis that revealed a markedly disturbed immune signature in the cerebrospinal fluid of the patients that is consistent with immune activation in the central nervous system, and a shift toward an allergic or T helper type-2 pattern associated with autoimmunity [112]. Later, the same author re-analyzed the cerebrospinal fluid from a patient cohort with CFS/ME and found suggestive patterns of disturbances in interleukin 1 signaling and autoimmunity-type patterns of immune activation in patients with atypical clinical characteristics [113].

Although the mechanisms underlying neuroinflammation in ME/CFS are unclear [104], it appears that positive feedback between the inflammatory state and the neuronal overactivation takes place [114]. On one hand, patients have to exert a significant effort to perform daily activities, which results in enhanced neural activation that leads to an increased production of pro-inflammatory cytokines, reactive oxygen species, and nitrogen species [65,66,106]. The central inflammatory component can also be triggered by the immunologic response to an initial infectious process [6]. Thus, the overwhelmingly increased inflammatory state cannot be countered by anti-inflammatory mechanisms, thereby encouraging the development of ME/CFS [115].

##### Neuronal Sensitization

Central sensitization is a characteristic of neuropathic pain that underlies chemical, functional and structural changes in the CNS [116]. It is manifested as an exaggerated response to noxious stimuli, a reduced threshold for pain, and spread sensitivity around the innervation territory of the injured nerve. It may be produced by the constant stimulation of inflammatory molecules, affecting peripheral sensitivity to further non-noxious stimuli [117]. Furthermore, sensitized neurons may continue to fire after the initial stimulation has ceased: a phenomenon that is referred as “kindling” [6]. Thus, an insidious peripheral-central neurogenic sensitization loop takes place, which would conceivably have the power to modulate the impact of symptoms in ME/CFS [118].

Peripheral pain can contribute to this sensitization by a process that manifest in hyperalgesia and contributes to ME/CFS symptomatology. This phenomenon takes place under long-term potentiation (LTP), in the context of temporal summation effected by repetitive nociception stimulation [119]. Thus, most of the pain symptoms displayed during ME/CFS may be produced by changes in synapse transmission in some of the primary structures involved in pain modulation such as serotonin and glutamate attenuation in encephalic pain modulation structures [117,120].

##### Glial Activation

The inflammatory response within the CNS favors a proper environment to cause peripheral sensitization. These nociceptive afferents not only interact with post-synaptic neurons, but they also trigger glial responses [104,121]. Calcium ion influx into glial cells, especially astrocytes, causes central terminals of the nociceptive pathway to release neuroactive-signalling molecules that activate the surrounding microglia [122]. These include the primary neuroexcitatory neurotransmitters such as glutamate, nitric oxide (NO), and potent pro-inflammatory cytokines such as tumor necrosis factor alpha (TNF-α) and IL-1B [6,107]. Activated microglia responds similarly to systemic inflammation by inducing superoxide and NO production. Superoxide and NO are free radical substrates of the peroxynitrite (ONOO-) radical, and hence sources of oxidative and nitrosative (O&NS) stress damage [123]. The enhanced glial activation results in neuroexcitation, neuroinflammation, and neurodegeneration through molecular alteration by oxidative stress, which is supported by neuroimaging studies that evidence a significant reduction in white and grey matter volumes in ME/CFS patients [124].

This glial activation due to systemic inflammation may be one of the causes of chronic pain in patients with ME/CFS, involving pathological processes of allodynia and hyperalgesia, via the impact of bidirectional neuroglial signaling [6]. Glia can be activated by neuronal stimulation and the inflammatory cytokines that they release may in turn link to neuronal glutamate receptors enhancing neuroexcitation [117,121]. Collectively, the overactivation of glia by neural and immunological signals can favor sickness behavior and peripheral symptoms that are observed in patients with ME/CFS.

Recently, overall research into glial activation due to systemic inflammation has been deepened. Some toxins have been described that are specifically capable of activating the glial cells called “gliotoxins”, which are produced by certain species of bacteria, fungi and viruses [125]. This activation could generate a gliopathy in the CNS. However, gliopathies have also been shown to be initiated by other molecules and neurotransmitters directly related to a systemic inflammatory state such as the pro-inflammatory cytokines TNF-α and IL-6, and high concentrations of glutamate and other pain-related neurotransmitters such as substance P [6,126].

#### 5.5.4. Alterations of Serotonin Transmission

Serotonin (5-HT) is a monoamine that is involved in the pathophysiology of many neuropsychiatric conditions that share some symptoms with ME/CFS, especially the extreme fatigue that does not improve with rest [127]. Central fatigue represents the contribution of the CNS to muscle fatigue; in other words, it is the progressive decline in the capacity to produce voluntary muscle force as exercise continues [128,129]. It is characterized by a decreased ability to contract muscle fibers adequately during motor activity, and is observed separately from muscle fatigue [129,130]. The cellular mechanism remains unknown, but the evidence suggests that central fatigue correlates with increased levels of 5-HT and its metabolites in the CNS [131,132].

It is known that the 5-HT1A receptor negatively regulates the activity of 5-HT neurons via the Gi protein, and is expressed as both a presynaptic autoreceptor on raphe neurons and as a significant postsynaptic receptor in the hippocampus, cortical, hypothalamic and spinal regions involved in mood, emotion, stress responses, and motor activity [127,128]. Unlike motoneurons excitation via the 5-HT 2B/C receptors, 5HT1A-mediated inhibition only occurred during prolonged stimulation of the dorsolateral funiculus and appeared to depend on the spillover of 5-HT to the initial axon segment, which does not receive serotoninergic innervation [133]. During high levels of release, 5-HT spills over to reach extra-synaptic receptor sites in the initial axon segment, and inhibits the generation of action potentials [130]. This prevents the hyperactivity of motoneurons, promotes motor unit rotations, and reduces detrimental muscle activity. All these findings have identified the 5-HT1A receptor as a critical contributor of central fatigue [129]. Thus, it is widely proposed that the obvious disability due to extreme fatigue may be because of punctual alterations in the serotoninergic transmission [134].

Several hypotheses aimed at defining the aetiology of these alterations have been proposed in the literature, but the ones that have more significant support at present are the consequences for the systemic inflammatory state and changes in the genotype of the proteins involved in the serotonergic pathway [135]. Inflammatory cytokines are known to act centrally to alter the metabolism and release of neurotransmitters including serotonin. For instance, inflammatory cytokines such as TNF-α and IL-1β acutely activate the serotoninergic transporter (SERT) through the stimulation of the p38 MAPK pathway and increase the concentration of other catecholamines in specific brain regions such as the anterior hypothalamus [110,136]. SERT transport serotonin from the synaptic cleft into the presynaptic neuron and together with 5-hydroxytryptamine transporter (5-HTT) is involved in the termination of the serotoninergic signaling [137]. Genetic studies reported specific polymorphisms in different components of the serotoninergic neurotransmission such as the 5-HT1A receptor and the serotoninergic transporter 5-HTT in patients suffering from ME/CFS [110,131].

It appears that the neuroimmune dysregulation occurring in patients with ME/CFS is capable of explaining the neuropsychiatric symptoms reported in this pathological condition. Thus, treatments toward the reduction of this inflammatory state and consequently neuroinflammation may in turn reduce the severity of the symptoms of this disease.

#### 5.5.5. Neuroendocrine

##### HPA Axis in ME/CFS

The interest in the role of the hypothalamic-pituitary-adrenal (HPA) axis in CFS developed from the careful observations that clinical conditions in which there is low circulating cortisol are characterized by debilitating fatigue [138]. Thus, Addison’s disease, glucocorticoid withdrawal, and bilateral adrenalectomy are all associated with fatigue and with other symptoms that are also seen in CFS, such as arthralgia, myalgia, sleep disturbance, and mood disorder [84,139]. These observations gave rise to the hypothesis that one of the features of the fatigue in ME/CFS is low circulating levels of cortisol [138]. 

Reviewing the literature, there is a wealth of studies that have conflicting conclusions about the dysfunction in the HPA axis in patients with ME/CFS. It seems that some of the reasons for the inconsistencies in the data include the heterogeneous nature of ME/CFS itself [30]. The hypofunction of the HPA axis as manifested by a low salivary cortisol-awakening response is the most replicated biological finding in ME/CFS adult patients [140,141]. Moreover, low baseline levels of HPA axis hormones; aberrant diurnal hormone variation; reduced HPA axis response to physical and psychological stressors; and enhanced sensitivity to glucocorticoids have been also reported in patients suffering from this disease [30]. Approximately half of the studies that have measured unstimulated cortisol and ACTH levels in blood or saliva, and others that determined the diurnal variation of urinary cortisol, reported some evidence for lowered cortisol levels at some point in the day in patients with ME/CFS [140]. However, there is no convincing evidence that any HPA axis disturbance is specific to ME/CFS, or that it is a primary cause of the disorder rather than being related to the many possible consequences or comorbidities of the illness [138].

One of the hypotheses that could explain the low cortisol levels reported in patients with ME/CFS is related to the nature of the dysregulation of the stress response. It is noteworthy to remark that since 1998, Scott et al. [142] have suggested the ME/CFS is a stress-related disorder. They hypothesized that initial stress might cause an elevation in corticotropin release hormone (CRH) with a consequent down-regulation of CRH receptors (CRHR) on the pituitary corticotrophs neurons. This down-regulation fails to normalize after the alleviation of stress or the subsequent reduction of CRH levels because of the abnormal plasticity in the CRH receptor. Thus, the hypofunctioning of the HPA axis is the consequence of a “stressed crash” or “exhaustion” phenomenon whereby the stress-induced HPA axis hyperfunction switches into HPA axis hypofunction following prolonged stress. Long-standing stress can result in an exhaustion of the stress response whereby the HPA axis is proposed to lose its ability to cope with environmental stress, coupled with decreased cortisol output [138,139]. Since plasma cortisol is mainly controlled by ACTH, there is a linear relationship between these two hormones in patients with ME/CFS [31,143]. 

Different interrelationships among hormones of the HPA axis, the sympathetic/adrenal medulla (SAM) system, and the thyroid system between ME/CFS patients and healthy controls have been reported in a number of studies [14,31,144]. This neuroendocrine imbalance affects the homeostasis of another major system that is involved in the pathophysiology of the disease: the immune system. It is well-known that the interaction between glucocorticoids and immune cells is crucial for the optimal development of the host immunological response [145]. There is bidirectional communication between immune-inflammatory pathways and the HPA axis. For example, HPA axis hormones exhibit negative feedback on the immune system, downregulating inflammatory responses, whereas pro-inflammatory cytokines stimulate the HPA axis to produce more HPA axis hormones [84,139].

Therefore, the findings in ME/CFS could be explained by at least two different mechanisms:

(a) Activation of immune-inflammatory pathways is secondary to HPA axis hypofunction by the attenuation of negative feedback of the HPA axis hormones on the immune system, and
(b) chronic activation of immune-inflammatory pathways play a causative role in HPA axis hypofunction [143].

In conclusion, the repeated activation of immune-inflammatory pathways in ME/CFS, including increased levels of pro-inflammatory cytokines, may be influenced by the HPA axis hypofunction [138]. Glucocorticoids have anti-inflammatory and adverse immune-regulatory effects by attenuating Th1 cell-mediated immune responses and promoting Th2-mediated and Treg functions [140,143].

Consequently, there are syntheses of cytokines and the activity of transcription factors, such as NF-κB, which modulates the viability of immune cells (e.g., monocytes and lymphocytes), impedes NO production, blocks promoter sites of pro-inflammatory genes (e.g., IL-1, IL-2, and IL-6), and activates anti-inflammatory genes such as IL-10. These glucocorticoid effects are mediated by binding to their glucocorticoid receptors (GCRs) in the immune cells [139].

Regardless of whether disruption of the HPA axis is primary or secondary, a more significant comprehension of the complexities of the pathophysiology of the ME/CFS has been gained by understanding the changes in the HPA axis. This knowledge could profoundly improve the symptomatic treatment of ME/CFS by adequately controlling the hormone dysfunction.

##### Hypocortisolism

Patients with ME/CFS show dysfunction of the HPA axis resulting in hypocortisolism, and an attenuated cortisol awakening response. Cortisol is the principal end product of the HPA axis and is involved in the regulation of several bodily systems. Two recent meta-analyses found that the cortisol awakening response (CAR) is the most common finding in the experience of fatigue, and may be relevant in the pathophysiology of the post-exertional malaise, which is a crucial feature of ME/CFS [146]. The CAR describes a surge in cortisol levels upon awakening and has two components:

(1) the total cortisol output within this period, and (2) the dynamic response, usually referring to the change in cortisol output from waking to peak levels [147].

Urinary sampling for cortisol is sometimes utilized in research designs, but offers only a summary index of cortisol production over a period [146,148]. Evidence from eight control studies indicated decreased within-person CAR and circadian cortisol variation within ME/CFS. However, a lack of validating studies prevents overly robust conclusions about the importance of these biomarkers in the pathophysiology of the CFS.

The cause of hypocortisolism in patients with ME/CFS remains unclear [145]. Some authors suggest that it may be caused by impaired central nervous system signaling of the adrenal glands, such as limited adrenocorticotrophic hormone (ACTH) output; decreased adrenal gland body size; a compensatory shift toward hypocortisolism after a period of HPA hyperactivity following chronic stress; and enhanced negative feedback of the HPA axis and reduced response of ACTH [148,149].

There is evidence suggesting that stressors, including emotional distress, may exacerbate ME/CFS symptoms, with fatigue being the most prevalent [138]. Emotional distress reactions, such as environmental challenges, may trigger physical symptoms in ME/CFS [150]. This finding has led to the proposal of a standard endocrinological pathway that may underlie the development of “stress-related” disorders, and which would potentially help explain common symptoms of enhanced stress sensitivity [141]. It can be concluded that using stress reduction interventions, such as perceived stress management skills in patients suffering from ME/CFS, could be useful to mitigate the symptomatology [143,148]. ME/CFS patients reveal a flatter cortisol awakening response, as well as a flatter diurnal slope of salivary cortisol output compared to healthy individuals [31], particularly in patients with early adverse life stressors [146]. It is plausible that persons with better stress management skills have less anxiety and negative mood, which in turn relates to lower evening cortisol levels and better HPA control over pro-inflammatory cytokine production [148]. 

## 6. Cortisol Treatments for Patients With CFS

Since the identification of endocrinological alterations in patients with ME/CFS, treatments with corticosteroids were assessed, most of them with positive effects in the symptomatology of the patients. Some studies that managed patients with ME/CFS with low doses of hydrocortisone showed favorable results in the reduction of symptoms, especially regarding a reduction in the fatigue levels in the short term [151,152].

## 7. Genetic Predisposition

As described previously, ME/CFS is a complex condition with a multifactorial aetiology involving multiple mechanisms [153]. As a focus of this review is to exhibit the impact of dysregulation of the neuro-immuno-endocrine relationships, we aimed at summarizing several findings reporting genetic polymorphisms in key neuroimmunoendocrine-related genes that might be involved in the ME/CFS condition. There are plenty of studies that have analyzed the gene expression in the peripheral blood of patients with ME/CFS, and have proposed candidates genes related to the risk of developing the disease [154,155,156,157,158,159,160]. Unfortunately, many of these studies did not confirm their finding with PCR, making some of the proposed results unreliable [161,162]. There are a few studies that have analyzed twins with the condition; however, a study conducted in 2001 using 146 female-female twin pairs with ME/CFS provided evidence that supported a familial aggregation of this syndrome [163]. More recent studies have proposed similar conclusions using principle components and latent class analyses to select genes that can have a heritable component, which is mostly involved in the HPA axis and cytokines [164,165].

A common approach to address the symptom heterogeneity of the disease in many studies is to subtype the patient population, allowing the research to focus on the analysis of the biological differences that underlie more specific manifestations of the disease [166]. Some authors attempted to subtype ME/CFS patients based on differences in gene expression profiles in order to approach particular etiological factors [162,167,168]. A symptom-based approximation of the disease has had success in identifying musculoskeletal, inflammatory, and neurological subtypes [169]. One of those studies enrolled patients with a defined ME/CFS phenotype and compared them to healthy blood donors using a microarray that represents the entire human genome and with quantitative PCR (qPCR) confirmation. They clustered the mean relative quantity of mRNA transcripts in patients with ME/CFS and identified seven subtypes of genes with similar profiles of expression. Relative quantification of the mRNA is an approach to determine the quantity of target mRNA in samples with a relation between them [170]. Analysis of mean age and sex ratios for each subtype revealed differences between males and females in the severity of the disease; differences in social functioning and emotional roles; and differences in the severity of individual symptoms between subtypes [162]. However, it is difficult to directly compare and interpret subtypes in various studies because of the differences in the study design, the subject selection, and the inherent variability of the data, among other differences.

The attempt to determine the genetic aetiology of ME/CFS is further obfuscated by diagnostic errors, phenotypic heterogeneity, and environmental effects [171].

### 7.1. Epigenetic Modification

Molecular studies using DNA methylation microarrays indicate that methylation plays an essential role in the regulation of several genes involved in the relationship with the pathophysiology of the disease [172]. The interaction between the environment and the development of the disease is highly supported, and may be explained by the epigenetic modification of some candidate genes [173]. This idea has been amply supported by an increasing number of studies that have carefully examined the epigenetic changes associated with the neuro-immuno-endocrinology axis of the disease pathophysiology [174,175,176]. The methylation of DNA is one of the most studied epigenetic modifications. It mainly occurs on the cytosines of the CpG dinucleotide sites across the genome, and regulates the gene expression without disrupting the nucleotide sequence, and may arise through genetic, stochastic, and environmental factors [176]. To date, there are plenty of studies relating the ME/CFS with epigenomic changes. De Vega et al. found significant differences in DNA methylation between CFS patients and healthy controls at 1192 CpG sites in 826 genes, with differential DNA methylation occurring in promoters, gene regulatory elements and within coding regions [175]. Most of these genes are involved in the adaptive immune response and the preservation of an inflammatory state [171]. It is known that epigenetic modifications are mechanisms that modify the long-term gene expression in response to an environmental stimulus [114,115,176,177]. Clearly, the infectious prodrome observed in a vast majority of cases of ME/CFS is indicative of the importance of this infectious exposure in genetic regulation. However, it is still not clear if the relationship between the epigenetic modifications of genes related to the adaptive immune response is the reason why individuals with ME/CFS are predisposed to contract viral infections, or if the infection is the necessary environmental stimulus for the epigenetic modification. Future research is required in order to correctly solve doubts about the epigenetic impact on ME/CFS.

### 7.2. Mechanisms

In the following section, an attempt will be made to demonstrate the genetic importance in the physiopathological mechanisms most supported in the literature. These mechanisms primarily include the neurotransmitter dysregulation, the alteration in the HPA axis, and the immune-inflammatory responses [178,179,180].

### 7.3. Neurotransmitter Dysregulation

The most common neurotransmitter alteration related to the ME/CFS in the literature is the serotoninergic system [153]. Some polymorphisms in the serotonin transporter, receptors, and synthetic enzymes are highly linked with ME/CFS. In a case-control study, Narita et al. identified a polymorphism in the serotonin transporter gene 5′ upstream region (5-HTTLPR) in ME/CFS patients, but not in controls [97,155]. Besides, in another case-control study, three were located in the 5-HT receptor subtype HTR2A (rs1923884, rs6311, and rs6313) and identified as associated with ME/CFS [180]. Also, two polymorphisms in the adrenergic signaling pathway were found to be more abundant in ME/CFS patients than in controls: the β2-adrenergic receptor and the catechol-O-methyl transferase (COMT) [87,181].

The enzymatic activity of COMT has been shown to be inversely related to the levels of catecholamines [182]. Considering previous studies that observed ME/CFS patients with elevated levels of norepinephrine and epinephrine, the COMT low-activity met/met genotype appears to be more prevalent amongst adolescents with ME/CFS [159]. 

Another neurotransmitter involved in disease pathophysiology is acetylcholine, although from a limited number of studies. A small sample study identified a cholinergic receptor SNP (mAChM3R) that featured prominently in ME/CFS patients, which is consistent with the alteration of NK cells in these patients [183]. Another study found antibodies against mAChM3R (a muscarinic receptor) in a population with ME/CFS, and in which a modest positive response occurred with reduced symptom presentation following the anti-CD20 intervention [87,184].

All of these studies have identified a genetic predisposition in the dysfunction of some central and peripheral neurotransmitters in patients with CFS/ME, which may explain much of the symptomatology of the disease, mainly related to the nervous system such as pain or muscle weakness.

### 7.4. Alteration in HPA Axis

In light of genetic analysis, a study identified that a genetic variation in POMC and NR3C1 might contribute to the pathophysiology of subgroups of patients with ME/CFS [184]. This finding is consistent with other reports of the association between the disease, and polymorphisms in NR3C1 [185]. NR3C1 is a glucocorticoid receptor gene that is influential in regulating the HPA axis function and blood glucocorticoid levels that have been highly related to the neuroendocrine pathophysiology of the disease [157].

### 7.5. Immune-Inflammatory RESPONSES

As previously described, immune dysregulation and the inflammatory reactions contribute to the pathophysiology of ME/CFS, and a genetic predisposition may contribute to the spread and persistence of a mild systemic inflammatory state. A study that investigated the human leucocyte antigen (HLA) class II alleles, and the receptor for advanced glycation end product (RAGE), found a significant association between HLA-DRB1 and multiple RAGE polymorphisms with the pathogenesis of ME/CFS [186]. Also, a previous study identified a clear association between the HLA-DQA1 alleles and the ME/CFS [187]. Although multiple studies have taken into account the existing relationship within the polymorphisms of cytokine receptors, none of them reported reliable and reproducible data with the disease [183,188].

## 8. Management

In the pharmacological approach to the management of ME/CFS, trials have had a poor external validity, and have proven to be inconsistent and inconclusive. The only systematic review of the pharmacological management of ME/CFS identified 20 drug therapies from 26 studies, and 18 applied the Fukuda criteria as the primary tool for inclusion criteria [189]. Eleven medications were shown to be either slightly, mildly, or moderately effective in their respective study groups. Outcomes were measured with clinician-administered and self-administered surveys or scales. From those 11 drugs, six of them had significant results in the fatigue outcome. This group included medications that were individually studied in clinical trials such as dextroamphetamine [190] and nefazodone [191], both with inconclusive results. Other drugs involved in the systematic review included rintatolimod, acetyl-l-carnitine, and intravenous immunoglobulin. Each one of these drugs has individual studies reporting some improvements in severe symptoms, especially in fatigue and cognitive impairment [192,193,194,195]. However, as stated earlier, none of them had the epidemiological significance to be the gold standard in the pharmacological treatment of ME/CFS. 

There are multiple mechanisms of action in the pharmacological therapies that are used for the management of ME/CFS. Dextroamphetamine is a well-known CNS stimulant and a sympathomimetic that induces the release of dopamine in the mesocorticolimbic circuits. Nefazodone is a serotoninergic modulating antidepressant, and acetyl-l-carnitine is an acid ester of carnitine that facilitates the movement of acetyl-CoA inside the mitochondria during the oxidation of fatty acids, with proven neuroprotective action [196]. There are medications whose mechanisms target a proposed pathophysiology of ME/CFS; one example is rintatolimod, which is an inducer of the interferon activity without helicase activation [192]. Rintatolimod has been studied due to its selectivity in its mechanism of action, its safety for the patient, and the initial success in open-label trials [197].

In the symptomatic management, the studies show results with meaningful outcomes. In the cognitive disability and functional status, Bonnet and Young respectively discussed the effects of moclobemide and lisdexamfetamine dimesylate [198,199]. However, the results given by Bonnet and Young were limited due to the questionnaires that were used to assess the cognitive and functional status and the sample used for the study. For fatigue and post-exertional malaise, some studies have evaluated low-dose hydrocortisone (5–10 mg daily), and found a short-term improvement in fatigue, but with a relapse of the symptoms once the drug is discontinued [152].

The fact that the pathophysiology of the ME/CFS is still not understood entirely opens the possibility of studying a number of non-pharmacological options, some of them with good results in the symptomatological relief [200]. One area of those non-pharmacological approaches is dietary interventions, including the mitoprotective diet, consisting of caloric restriction, fasting diets, and ketogenic diets [201]. The mitoprotective diet has a crucial role in the regulation of the mitochondrial dysfunction due to oxidative stress, which is a well-known pathophysiological feature [202,203]. The impairment of the energetic balance due to reduced mitochondrial capacity in the skeletal muscles of ME/CFS patients has mainly been studied and, as previously seen, is one of the hallmarks of the disease. This dysfunction can be attributed to many triggers, including chronic viral infections; these infections have the potential to create a cycle that disrupts the mitobioenergetics of affected cells, increasing the oxidative stress damage by alteration of the anti-oxidants components of the cell [204]. The improvement of this mitochondrial dysfunction, and the subsequent O&NS damage, is shown to have an inverse relationship with fatigue severity scores in patients with ME/CFS [205]. With this background, the caloric restriction of the diet can lead to adaptive responses that affect the inflammatory pathway, the energy metabolism, the DNA repair and the modulation of the O&NS [206]. However, the exact mechanism of this diet is not well understood. Along with mitoprotective diets, natural antifatigue supplements have also had promising results in animal models enhancing exercise tolerance [207].

One of the constituents of mitoprotective diets is the ketogenic diet, which is defined as a high-fat, deficient carbohydrate diet that mimics the effects of caloric restriction or the fasting diets. The ketone bodies acetoacetate and β-hydroxybutyrate are produced during lipolysis through the generation of acetyl-CoA, as occurs in a fasting state or with a minimal intake of carbohydrates. The mitochondrial effects of the ketogenic diet are similar to those observed during the caloric restriction by a similar mechanism as described earlier. In experimental studies with animals, it has been found that the β-hydroxybutyrate is an endogenous and specific inhibitor of class I histone deacetylases, resulting in global changes of genes transcription, including the ones involved in the oxidative stress resistance factors [208,209]. However, the effects of the ketogenic diet are not all beneficial for the patient, and may not contribute to the complete recovery of the disease, as ME/CFS has other pathophysiological pathways, as described herein.

There is also literature that demonstrates mitoprotective roles in specific medications, such as sodium dichloroacetate, which enhances the activity of the mitochondrial enzyme pyruvate dehydrogenase [210]. However, it has been seen that this medication does not work in all patients [211], thus generating more doubts about the hypothesis of whether mitochondria is the only etiology of the disease.

There are other trials and reviews of pharmacological and non-pharmacological therapeutic option for ME/CFS; most of these are directed toward the reduction of the inflammation and the oxidative stress with the objective of symptomatologic relief [219]. Table 3 briefly summarizes the current therapeutic approaches that are being studied. That is the case of the probiotic interventions showing good outcomes in the management of gastrointestinal symptoms observed in the ME/CFS, reducing the cytokine levels entering the systemic circulation by the leaky gut phenomena [220]. However, as for most of the therapeutic options, the studies have inconclusive results due to limited data and validation.

## 9. Discussion

In this comprehensive review, a holistic approach to a new disease has been presented. It is evident that diagnostic tests for clinical practice are not specific to the condition, which is reflected in the high rates of underdiagnoses (85–90%). When reviewing the historical perspective of ME/CFS, the relevance of infectious prodrome in the understanding of pathophysiology was identified. The first clinicians to describe the syndrome, as reviewed earlier, immediately associated the disease with an ongoing infection. Nowadays, with a significant core of research, it is known that the disease has its pathophysiological sustenance in “three pillars” that continuously interact with each other: the immune system, the nervous system, and the neuroendocrine network. Table 1 offers a brief summary of the main features of the tissues involved in the “three pillars” hypothesis. As can be seen in Table 3, current therapeutic strategies target several elements of the proposed neuro-immunoendocrine network, which also supports the “three pillars” hypothesis that we are discussing in this review. 

The immune system is involved in modulating neural plasticity, learning, and memory, although the precise link between these two seemingly distinct systems was, until recently, unclear [54]. The connection may be explained by the coevolution of the nervous and immune systems, as the two systems share mechanisms of stimulation, cell communication and signaling, gene regulation, and supracellular organization. The immune system supports the central nervous system (CNS) and aids functional recovery by facilitating the renewal, migration and cell lineage specification of neural progenitor cells [221]. 

The immune system is involved in the stress response, since stress activates the immune system, leading to peripheral inflammation that may ultimately contribute to the onset of a part of the symptomatology of the disease [222]. Indeed, stress has been shown to be an essential predisposing factor in the development of several neurodegenerative and psychiatric disorders [223]. The hypothalamic–pituitary–adrenal (HPA) axis and the systemic sympatho-adrenomedullary (SAM) system are essential modulators of stress response systems [224]. The HPA axis is an endocrine pathway that regulates standard stress response and merges with the immune system to maintain homeostasis [138,139]. Therefore, stress stimulates the release of glucocorticoids, particularly cortisol, which is able to cross the BBB and alter the transcription of proteins in the brain [225]. Glucocorticoids bind to the glucocorticoid receptor (GR), resulting in disassociation from the heat-shock protein, and promoting a structural change of the receptor that enables the glucocorticoid-GR complex to enter the nucleus. The glucocorticoid-GR complex binds to the glucocorticoid response element on the DNA, resulting in the activation of transcription of immune-mediator genes, among others [223,226]. Therefore, stress hormones, such as cortisol, have the ability to regulate the immune system. However, HPA is not the only neuroendocrinological network that can interact with the immune system. The SAM is also activated by stress, leading to the release of catecholamines (e.g., epinephrine and norepinephrine) in the adrenal medulla in response to stress [134,227]. Catecholamines have been found to regulate the synthesis of immune system mediators through β-adrenergic receptor stimulation [226], suggesting an alternative pathway that links the neuroendocrine and immunological systems. 

ME/CFS patients show heightened negative feedback inhibition of the HPA axis, which is associated with hypocortisolism and heightened GR sensitivity [224]. As a result, patients with ME/CFS often show heightened immune responses owing to the combined effects of chronic stress with activated microglia [130,223] and increased HPA-axis sensitivity [224]. The HPA axis has been of great importance for the understanding of the pathophysiology of the disease, since the consequences of its alteration, such as hypocortisolism, have allowed us to understand the persistence of an altered immune status, the high risk of infections and the generation of humoral autoreactivity.

Although the metabolic sphere is not part of the aetiopathological pillars of the disease, it is clear that it is a physiological aspect compromised in patients with ME/CSF. The dysregulation of the energetic metabolism can be understood as the tip of the iceberg, which will trigger the symptomatology experienced by the patient. However, the etiology of this metabolic imbalance in ME/CSF has not yet been understood, which is most likely because it is a pathological process that is the product of complex multisystemic interactions. Studies on metabolism and CFS suggest irregularities in energy metabolism, amino acid metabolism, nucleotide metabolism, nitrogen metabolism, hormone metabolism, and oxidative stress metabolism [228,229]. The overwhelming body of evidence suggests an oxidative environment with the minimal utilization of mitochondria for efficient energy production, leading to thoughts of some type of etiology in this organelle, but as we have seen previously, apparently the mitochondria are affected with the course of the disease [230]. As well as throughout the review, more studies are needed to understand which is the metabolic pathway that is first affected or which is the most altered in order to understand where to direct the etiological search in this complicated disease.

ME/CFS remains a challenge for the biomedical community. Pathophysiological research should follow two strategies. The first consists of distinguishing ME/CFS from other disorders. The characteristic pathophysiology of chronic fatigue in neurological disorders, or during cancer, or in inflammatory diseases such as rheumatoid arthritis, should be compared with the fatigue in ME/CFS. The second strategy consists of investigating the similarities and dissimilarities in functional somatic syndromes. Modern neurosciences offer some explanatory models, which might bridge the gap between somatic and psychological models for ME/CFS and other functional somatic syndromes [225].

Due to the unclear aetiology, diagnostic uncertainty, and the resultant heterogeneity of the ME/CFS, there are no established treatment recommendations in the clinical practice [32]. Systematic reviews have investigated the effectiveness of several ME/CFS treatments [231,232]. Cognitive behavior therapy (CBT) and graded exercise therapy (GET) are some of the few interventions that are proposed as beneficial in improving quality of life [233], but these remain controversial and have been recently criticized by others [234].

In practice, pharmacological or non-pharmacological treatments have been directed toward relieving symptoms and improving quality of life [32]. Table 3 shows some of the treatments that are being studied for the relief of symptomatology in patients with CFS/ME. Several randomized control trials have shown improvement in the most disabling symptoms with the use of certain drugs [189,213,218,231,235]. Additionally, there are studies that have proven significant improvement of the symptomatology using a drug together with an adjuvant; this is the case of the use of the selective inhibitors of the reuptake of serotonin, which were studied alongside Dengzhanshengmai capsules, which are a traditional Chinese medicine, resulting in a significant improvement in general fatigue, as well as in mental and physical health [214]. However, it should be clarified that more studies are required to reproduce these conclusions and results in order to provide more scientific sustenance so that a pharmaceutical indication can exist. There has also been insufficient evidence of the effectiveness of pharmacological, supplementary, complementary, and other interventions [32,54]; treatment with anticholinergics, hormones, nicotinamide adenine dinucleotide, and antidepressants have been studied without conclusive results [236,237]. Similar to many patients with other chronic diseases for which conventional medicine has been unable to provide relief, those with ME/CFS use alternative treatments with unknown outcomes. These treatments include megavitamins, energy healing, herbal therapies, and special diets [219]. However, controlled studies to clarify the real effectiveness of these therapies do not exist.

Longitudinal studies of varying duration have shown that although 17–64% of the patients with ME/CFS improve, less than 10% fully recover, and another 10–20% worsen during follow-up [238]. Older age, longer illness duration, fatigue severity, comorbid psychiatric illness, and a physical attribution are some risk factors that worsen the prognosis of the patient [46]. As expected, children and adolescents appear to recover more rapidly and tend not to have recurrences of the disease in the future.

During this review we have seen that various biomarkers have been chosen to explain many of the pathophysiological processes involved in the aetiology of the disease; nonetheless the results of the different studies on these biomarkers are inconclusive, and do not define a clear pathological process. This review proposes a physiopathological hypothesis, using many of the results that have emerged from the molecular studies of ME/CFS. Although the scientific community has come to propose reproducible molecular bases, only the molecular explanation of some of the symptomatology of the disease has been achieved. For example, the biomolecular reason for fatigue and lack of energy has clearly been explained by many authors, such as Rasa et. al., proposing both a mitochondrial dysfunction and an alteration in the use of energy by myocytes [239]. However, in this example, the mitochondrial alteration does not necessarily explain the totality of the symptomatology and the aetiology of this alteration is still not clear. Other studies have proposed an infectious aetiology; however, as with other attempts, not all patients that have ME/CFS have an infection in the onset of the disease [42,240]. Taking this into account, we can conclude that the studies on the biomarkers involved in the disease present frustrating results for the research groups that aim to understand the biomolecular bases of the disease in order to generate strategies for effective treatments.

More knowledge about the psychoneurobiology of ME/CFS and the natural history of the disease is needed to improve our understanding of this illness, and thereafter to allow the development of more effective treatments that can significantly improve the quality of life for patients, and lead them to recover their functionality in the shortest possible time.

## Figures and Tables

**Table 1:** Features and function of main tissues representing the three pillars of myalgic encephalomyelitis/chronic fatigue syndrome (ME/CFS). HPA: hypothalamic–pituitary–adrenal.

System	Tissue/Cell	Feature	Ref.
Central Neurological System	Neuron	The symptomatology is related to a variety of sources of chronic neurological disturbance and associated distortions and chronicity in noxious sensory signaling and neuroimmune activation	[9]
	Glial cells	There is a significant blood–brain barrier permeability, microglia activation through toll-like receptors (TLR) signaling, secretion of IL-1B, upregulation of 5-HTT in astrocytes, reduced extracellular 5-HT levels, and hence a reduced activation of 5-HT receptors	[10]
Immune System	Lymphocytes Th1/Th2	Significant bias toward Th2 immune responses in CFS patients leading to an effector memory cell bias toward type 2 responsiveness	[11]
	NK cells	Reduction of cytotoxic activity in CFS, leading to a higher susceptibility of infection	[12]
	B cells	Persistence of autoreactive cells that can generate autoantibodies during common infections	[13]
Endocrine System	Hypothalamus–pituitary–adrenal (HPA) axis	Enhanced corticosteroid-induced negative feedback, basal hypocortisolism, attenuated diurnal variation, and a reduced responsivity to challenge	[14]

**Table 2:** Common symptom range and key features of myalgic encephalomyelitis/chronic fatigue syndrome patients (ME/CFS).

Symptom	Description	Reference
Fatigue	Fatigue is not the result of ongoing exertion, is not relieved by rest, and is medically unexplained. Fatigue can worsen with prolonged upright posture or even low-energy consumption tasks.	[50]
Sleep Dysfunction	Sleep is unrefreshing with disturbed quantity or rhythm that can include daytime hypersomnia, night time insomnia, and day/night reversal.	[3]
Muscle Pain	Muscle pain is more common in the pediatric population and can be explained by a comorbid fibromyalgia.	[51]
Joint Pain	Joint pain is not a common condition and can be related to autoimmune comorbidities.	
Cognitive Dysfunction	Slow mental processing speed, impaired working memory, poor learning of new information, difficulty with word retrieval, increased distractibility, decreased concentration and attention span, and inability to multitask; all of which are collectively described by patients as “brain fog”.	[52]
Headaches	Frequently, patients suffer chronic, daily, new onset headaches, which can fluctuate in severity from week to week. If they are episodic, a diagnosis of migraine should be considered.	[3]
Post-Exertional Malaise	Normal activity or moderate exertion is followed by worsening of malaise, intense fatigue, and other symptoms. Recovery is difficult for the patient and usually takes more than 24 h.	
*One of the following:* (a) Autonomic Manifestations (b) Neuroendocrine Manifestations (c) Immune Dysfunction	Autonomic manifestations: orthostatic hypotension, exercise intolerance, sweating abnormalities digestive, urinary and sexual alterations. Neuroendocrine manifestations: Tolerance for stress, anxiety, or panic attacks, anorexia, recurrent feeling of feverishness Immune manifestations: Tender lymphadenopathy, sore throat, new sensitivities to food or medications.	[26]

**Table 3:** Current therapeutic strategies for ME/CFS.

Medication	Examples	Intervention	Adverse Reactions
NSAIDs	Ibuprofen, Naproxen	Relieve frequent or severe joint and muscle pain, headaches, reduce fevers and inflammation [212].	Gastrointestinal distress and bleeding
Tricyclic antidepressants	Amitriptyline, Doxepin, Nortriptyline, Desipramine	Symptom relieve, improve sleep, and relieve pain in much lower doses than those used to treat depression. Has anti-anxiety effect and improve locomotor activity [213].	Sedation, urinary retention, sexual dysfunction, weight-gain comorbidities.
Selective serotonin-reuptake inhibitors	Fluoxetine, Sertraline, Paroxetine	Helpful for anxiety/depression and other mood disorders in patients with ME/CFS, as well as patients with chronic neuropathic pain [214].	No specific adverse reactions have been described in the RCT
Antiviral Drugs	Rintatolimod, Valganciclovir	Enhance the NK-function and influence the 2-5A-synthetase pathway, producing an objective improvement in exercise tolerance and a reduction in ME/CFS-related concomitant medication usage [193,215].	Is a well-tolerated medication in the right dosage
Monoclonal Antibodies	Rituximab	Decrease the activity and number B-cell by inhibiting CD20, thus reducing inflammation. Studies demonstrate symptoms alleviation and improvement in quality of life within a 12-month follow-up [216,217].	Neutropenia, and increase of severe infections
Complementary and alternative medicine	Nutritional supplements, Acetyl-l-carnitine, Essential fatty acids, Magnesium, Vitamins, Coenzyme Q10 plus	Nutritional supplements may improve ME/CFS-related physical and mental fatigue in patients with specific nutritional deficiencies [215]. There are discrepant results in most of the RCT, and further research is needed in order to conclude a specific therapeutic role.	No specific adverse reactions have been described in the RCT of nutritional supplements
Corticosteroids	Hydrocortisone, Frudocortisone	Associated with statistical improvement in ME/CFS symptoms, especially in physical fatigue [218].	Adrenal suppression, mood disorders, weight-gain comorbidities.

See Reference [219] for additional details.
